# Self-control enhances vigilance performance in temporally irregular tasks: an fNIRS frontoparietal investigation

**DOI:** 10.3389/fnrgo.2024.1415089

**Published:** 2024-09-18

**Authors:** Salim Adam Mouloua, William S. Helton, Gerald Matthews, Tyler H. Shaw

**Affiliations:** Center for Excellence in Neuroergonomics, Technology, & Cognition, Department of Psychology, George Mason University, Fairfax, VA, United States

**Keywords:** self-control, fNIRS, temporal irregularity, vigilance decrement, frontoparietal networks, stress, mental resources, neuroergonomics

## Abstract

The present study investigated whether trait self-control impacted operators' behavior and associated neural resource strategies during a temporally irregular vigilance task. Functional near-infrared spectroscopy (fNIRS) readings of oxygenated hemoglobin (HbO2) and deoxygenated hemoglobin (HbR) from 29 participants were recorded fromthe prefrontal and parietal cortices. Self-control was associated with better perceptual sensitivity (A') in the task with the irregular event schedule. A left-lateralized effect of HbO2 was found for temporal irregularity within the dorsomedial prefrontal cortex, in accordance with functional transcranial doppler (fTCD) studies. Self-control increased HbR (decreasing activation) at right superior parietal lobule (rSPL; supporting vigilance utilization) and right inferior parietal lobule (rIPL; supporting resource reallocation). However, only rSPL was associated with the vigilance decrement—where decreases in activation led to better perceptual sensitivity in the temporally irregular task. Additionally, short stress-state measures suggest decreases in task engagement in individuals with higher self-control in the irregular task. The authors suggest a trait-state-brain-behavior relationship for self-control during difficult vigilance tasks. Implications for the study include steps toward rectifying the resource utilization vs. allocation debate in vigilance—as well as validating HbO2 and HbR as effective constructs for predicting operators' mental resources through fNIRS.

## 1 Introduction

Vigilance involves extended attention directed at a wide variety of real-world tasks such as monitoring in air-traffic control, driving, studying, surgery, and many more (Parasuraman et al., 1987). General characteristics of a vigilance task involve an inducement of decrement over time, passive observation, and detection of stimuli that are imposed upon the operator. Crucially, the length of such tasks is highly variable—ranging from as short as 12 min to several hours or longer (Temple et al., 2000). The concept has been studied for decades in applied experimental psychology (Davies and Parasuraman, 1982; Warm et al., 1992; Szalma et al., 2004; Shaw et al., 2016; Wiese et al., 2019). Vigilance performance is most often explained in terms of cognitive resource theories of performance in which the decline in performance typically seen in vigilance tasks is attributable to a loss of cognitive resources available for performance (Warm et al., 2008). There are several lines of research that support the resource position. First, subjective reports offered by vigilance observers consistently show that vigilance performance is affected by the psychophysical demand placed upon observers, such that task performance tends to vary inversely with cognitive load (Helton et al., 2005; See et al., 1995; Shingledecker et al., 2017). Second, numerous studies using the NASA Task Load Index (NASA-TLX; Hart and Staveland, 1988), a self-report rating scale that provides a measure of perceived mental workload, have indicated that the performance of vigilance tasks requires a substantial degree of mental effort (Warm et al., 2008; Satterfield et al., 2012; Finomore et al., 2013; Monfort et al., 2018).

Lastly, and perhaps most importantly, research exploring the neurophysiological underpinnings of vigilance supports a resource model of vigilance. While there are older investigations using functional magnetic resonance imaging (fMRI) and electroencephalography (EEG; see Parasuraman et al., 1998 for a review), most of the recent studies involve transcranial doppler sonography (TCD). The TCD studies point out that the decline in performance over time most characteristic of vigilance performance is mirrored by a similar decrement in neurophysiological activity, pointing to a depletion of mental resources (Shaw et al., 2019).

In recent years, researchers have posed a call to action for better quantification of renewable mental resources in the brain (Dehais et al., 2020; Mouloua et al., 2022; Helton and Wen, 2023). Such a mission would yield objective findings on the extent that resources are “drained” during the course of vigilance, “placed elsewhere,” or a combination of both (Helton and Warm, 2008). From an idiographic perspective, predicting individual operators' outcomes is critical to prevent real-world lapses in vigilance. For example, there may be more resources available to a highly-performing individual, or better allocation of those resources to the task instead of exogenous and endogenous distractions. Importantly, which traits predict performance and mental resource strategies?

One possibility that has recently been explored is that self-control influences performance (Becker et al., 2015; Satterfield et al., 2019; Harwood et al., 2024). Trait self-control is related to one's general disposition in life toward regulating common habits such as time management, spending choices, and academic outcomes (Tangney et al., 2004). In Tangney's trait self-control model, the construct governs one's overarching propensity for excitatory and inhibitory regulation (e.g., working toward long-term goals vs. avoiding interrupting others). Exerting state self-control, on the other hand, is an individual's attempt to change the way they would otherwise think, feel, or behave (Baumeister et al., 1994; Muraven and Baumeister, 2000). In Baumeister's self-control or self-regulatory strength model, it is suggested that exercising state self-control consumes self-control strength which is dependent upon resources. If these resources are drained, there are fewer resources available for subsequent tasks that would require self-control. Previous studies have shown that performing acts such as controlling one's emotions (Muraven et al., 1998), or resisting tempting foods like cookies (Baumeister et al., 1998) leads to poorer performance on a subsequent test of state self-control. When it comes to vigilance, there is some evidence that suggests that dietary restriction leads to poorer performance on vigilance tasks (e.g., Green et al., 1994, 1995). However, the evidence that state self-control impacts vigilance is murky at best (Satterfield et al., 2019). Moreover, meta-analyses suggest no ego depletion effect for attention (Carter et al., 2015), and more recently—no ego effect for attention or working memory tasks but an effect for emotional videos (Dang, 2018). Given the interplay between excitation and inhibition needed in a vigilance task (catching rare signals vs. avoiding false positives), perhaps one's innate capacity for self-control is more relevant than self-control depletion. Therefore, how does the inherently more stable construct of trait self-control relate to performance? In our study, we sought to determine whether high trait self-control was associated with more efficient resource utilization and allocation, as well as task performance.

An examination of individuals with high self-control and the associated brain mechanisms tells a different story than the Satterfield et al. (2019) study. Becker et al. (2015) examined the relation between self-control and vigilance but also included fTCD recordings. fTCD is an ultrasound technique used to examine cerebral blood flow velocity in the middle cerebral arteries. There is an abundance of evidence that suggests that the fTCD measure can successfully index the utilization and allocation of cognitive resources during vigilance (see Shaw et al., 2019 for a review). While the results of that study did not show differences in performance between high and low self-control observers (although there was a marginally significant trend favoring the high self-control group), results relating to the neurophysiological measure showed that while there was a decline in blood flow velocity in the low self-control group, there was no such decrement in the high self-control group. This points to the likelihood that high self-control individuals have superior allocation strategies during vigilance. Perhaps one explanation for the lack of a performance effect when exploring the self-control-vigilance relation is that the vigilance contexts previously explored did not require self-control. For example, in both the Satterfield and the Becker studies, the task used was a short 12-min vigilance task that was temporarily regular and somewhat predictable. Perhaps having high self-control will better serve an observer in situations where the schedule of events is irregular. Previous studies that have explored the irregularity of background events has shown inferior performance as compared to tasks with regular events. The theory that has been proposed to explain this phenomenon, the event asynchrony effect, suggests that since observers cannot be certain when an event requiring detection will occur that they must better govern the mechanisms for attention required to monitor the display (Shaw et al., 2012: Scerbo et al., 1987).

Here we propose that trait self-control alters the rate of vigilance decrement through: (1) adaptation to task demands over time (i.e., task engagement; see Matthews et al., 2011 for a comprehensive overview of this), through increased or decreased (2) utilization and (3) allocation of vigilance resources (dorsal and ventral attentional network activation). Broadly, we suggest that vigilance (McEwen and Wingfield, 2010) induces differences in behavioral and biological adaptation that are predicted by trait self-control. Utilizing a multidimensional measurement of stress (Hitchcock and Matthews, 2005) is crucial for this purpose, as we can delineate types of stress adaptation (cognitive, affective, energetic).

The purpose of the current study is to examine the relation between trait self-control and vigilance when the task is temporally irregular. Furthermore, we used functional near-infrared spectroscopy (fNIRS) to examine the neural underpinnings of performance. Previous studies examining the relation between self-control and performance have revealed mixed results. For example, Satterfield et al. (2019) investigated the relation between state self-control, trait self-control, and vigilance performance. In that study, the authors attempted to deplete self-control using a typing task that had been previously demonstrated to successfully deplete state self-control in previous studies (Rieger, 2004; Muraven et al., 2006). Moreover, trait self-control was assessed using the Tangney, Baumeister, and Boone Self-Control Scale (Tangney et al., 2004). The results of that study revealed no relation between self-control and vigilance, even when self-control was presumably depleted in one of the conditions. Alternatively, a study by Becker et al. (2015) revealed that trait self-control was related to more efficient resource allocation as measured by functional Transcranial Doppler Sonography (fTCD). Clearly, more research is needed in this area to reconcile these findings.

Attention is driven by complementary frontoparietal networks in the brain that capture critical subprocesses of attentional maintenance. The dorsal frontoparietal network involves right medial frontal gyrus (rMFG) and right intraparietal sulcus (rIPS; in our study measured via superior parietal lobule) and is involved in top-down processing, while the ventral frontoparietal network involves rMFG and right temporoparietal junction (rTPJ; in our study measured via inferior parietal lobule) and is involved in bottom-up processing (Corbetta et al., 2008; Painter et al., 2015). Meta-analyses of brain systems in vigilance suggest the interplay between the dorsal and ventral frontoparietal networks is crucial (Langner and Eickhoff, 2013). More specifically, those results show rTPJ is involved in reorientation signaling, rIPS is involved in attentional priority signaling, and medial prefrontal regions are involved in “re-energizing.” The existence of distinct systems subserving these purposes is well-supported (Posner and Petersen, 1990). Furthermore, research indicates that the frontoparietal networks intersect and communicate via right medial frontal gyrus rMFG (Drummond et al., 2013). However, does a decrease in either dorsal or ventral network utilization possibly reflect resource strategy efficiency?

Self-control might play an important role in the underlying cognitive control structures associated with sustaining attention. Schneider and Chein (2003) have modeled an extensive system of the latter mechanisms through the frontoparietal networks. The control processing system mainly involves elements of the dorsal attention network, including DLPFC, posterior parietal cortex (which includes IPS), medial temporal lobe (MTL), and the anterior cingulate cortex (ACC). They note that “learning appears to be a direct function of the number of controlled processing executions, with little impact of automatic transmissions” (Fisk and Schneider, 1984; Schneider and Chein, 2003). Thus, if the efficacy of controlled processing can be influenced by self-control, then it might explain how some people perform better when tasks are too difficult to adapt to. Stress state profiles support the notion that active fatigue occurs in the case of controlled processing (Hitchcock and Matthews, 2005). Therefore, we suggest changes in state task engagement to be sensitive to individual differences in self-control. Aston-Jones and Cohen (2005) found that attentional shifts governing engagement are mediated by TPJ signals coming from MTL, toward rMFG and right inferior parietal lobule (rIPL). This foundational narrative, when combined with the controlled processing network model (2003), reconciles the missing puzzle piece along the path between subcortical and posterior parietal regions. Thus, in a vigilance framework where learning does not occur, we might expect TPJ to perform the opposite role—suppressing those attentional shifts instead. In posterior parietal cortex and IPS, activity decreases as performance increases (similarly to dlPFC and ACC; see Chein and Schneider, 2005). Thus, we should expect that the dorsal network as a whole is relevant to interpreting performance changes in vigilance. Olesen et al. (2004) found that several weeks of working memory training using a variably-mapped task also increased activation in the dorsal network (MFG and IPS). This is consistent with a long-scale vigilance decrement, because a consistently-mapped task was not used. Since vigilance leads to increased energy expenditure, we suggest self-control will show the largest performance benefits on variable mappings (unpredictability). The distinction between consistent and variable mappings is analogous to temporally regular and irregular events, respectively.

While the neutral events in vigilance tasks usually do not require an overt response by participants, the schedule of these events can nevertheless have an overt impact on monitoring behavior. Most vigilance tasks that occur in the wild will not have a consistently mapped regular schedule of events but will more than likely follow an irregular pattern. This temporally irregular scheduling of events has been investigated in prior research. In a previous study exploring regular and irregular event schedules, Shaw et al. (2012) found that individuals exhibited the trademark behavioral decrement over time for both regular and irregular events. However, performance was worse in the condition where the event schedule was irregular instead of regular, a finding consistent with other research (Scerbo et al., 1986, 1987). Critically, it was revealed that cerebral blood flow velocity (CBFV) as measured by functional Transcranial Doppler Sonography (fTCD) displayed effects of event schedule and time on task and these effects were moderated by cerebral hemisphere. Hemovelocity declined more steeply in the right hemisphere as compared to the left hemisphere (consistent with the typical finding that there is right-hemispheric dominance in vigilance; Parasuraman et al., 1998), but the left hemisphere indicated irregularity-specific declines in performance, while the right hemisphere exhibited declines in both event schedules. The authors suggest that bilaterality of activation in the brain during vigilance increases with higher expenditure of cognitive resources, a finding consistent with other research (Helton et al., 2010; Shaw et al., 2016; Harwood et al., 2017).

## 2 The current study

In the present study, we used fNIRS to re-examine the self-control and vigilance relation, but specifically in contexts where event presentations are either temporally regular or temporally irregular. Utilizing fNIRS affords superior spatial resolution to previous vigilance studies using fTCD, because near-infrared light's absorbance in the cortex allows for examination of functional subregions of activation anywhere in the superficial cortex layers (i.e., right orbitofrontal cortex or right superior parietal lobule). This is advantageous over fTCD, which is only able to determine activation via the left- vs. right-hemispheres. Additionally, because fNIRS is fully portable, we are now able to map functional networks in the brain in highly ecological contexts such as operational environments (Curtin and Ayaz, 2018). This allows for a novel conception of cognitive resources via more than one dimension, including supply (oxygenated hemoglobin, HbO2) and demand (deoxygenated hemoglobin, HbR) dynamics. Given the difficulty in ascertaining what exactly the “mental resource” is, fNIRS might be particularly useful for researchers studying vigilance. Lastly, fNIRS has high enough temporal resolution to characterize vigilance responses at a trial level as opposed to a block level, which enhances its use case for examining the temporal regularity of event schedules beyond that of fTCD.

Previously, accounts of resource theory have often focused on the right prefrontal cortex. Examinations of this region have typically been justified by its connections with norepinephrine systems (Parasuraman et al., 1998). However, the parietal regions play a more salient role in sensory-related motivations and serve as a check on prefrontal systems (i.e., feelings overriding motivations). Critically, ventral parietal regions mediate the influence of norepinephrine (NE) on attention and thus might clarify the role of resource utilization or reallocation in vigilance (Aston-Jones and Cohen, 2005; Bouret and Sara, 2005; Dehais et al., 2020). When these regions are deactivated (via surges of noradrenaline), vigilance-related processing increases. Therefore, we suggest two biomarkers of vigilance resources that may be practically useful in cases where operators experience performance decrements. First, we define increased dorsal network activation as the means by which an operator maintains attention (vigilance is hard work) via an optimal level of resource utilization (Warm et al., 2008). Within the context of fNIRS, using too many resources (resource inefficiency) would be taxonomized by an increase in activation due to saturating blood oxygen in the dorsal network; this is opposed to a decrease in cerebral blood flow velocity seen in fTCD studies (Mouloua et al., 2022). Second, we define increased ventral network activation as the means by which an operator's attention naturally shifts toward task-unrelated thoughts and distractions in the environment throughout a vigil (vigilance is also boring; and on the latter point, see Scerbo, 2001). Mismanaging resources (resource misallocation) would be taxonomized by an increase in activation within the ventral network. Using this framework, resource theory and mindlessness theory can coexist and be empirically examined as two dimensions within the same study (vigilance is hard work and boring; see Dehais et al., 2020). One solution to the decrement may be lowering the amount of resources utilized by an operator; decreasing dorsal network activation. Alternatively, we might be able to mitigate the operator's propensity to become distracted by their environment or internal dispositions via resource reallocation; decreased ventral network activation. In our study, we sought to determine to what degree resource utilization and resource reallocation are present, as well as whether both theories play a role in the behavioral decrement—via the dorsal and ventral networks, respectively. Crucially, we sought to investigate whether an operator's ability to regulate themselves (trait self-control) influences these metrics.

It is hypothesized that self-control will modulate vigilance performance, but specifically in contexts where event presentations are irregular. More specifically, we predict superior performance of high self-control individuals in the irregular condition as compared to the regular condition, due to the increased need to inhibit responses under temporal pressure. Moreover, we predict that high self-control individuals will show increased neural efficiency (Becker et al., 2015) consistent with previous evidence suggesting that self-control influences the allocation of information processing resources. Taken together, these findings will have implications for resource theories of vigilance and the selection issue that has long been a concern of vigilance researchers (e.g., Finomore et al., 2009; Shaw et al., 2010; Matthews et al., 2011; and see Table 1). Specifically, we predicted that:

Prediction 1: Participants will perform better in the condition where the event schedules are regular as compared to irregular. This will be reflected in a higher hit rate and a lower false alarm rate.
Prediction 2: Self-control will enhance vigilance performance in the irregular task. More specifically, we hypothesize that individuals who score higher on trait self-control will have higher hit rates and lower false alarm rates.
Prediction 3: Self-control will have a greater moderating impact on watch periods when the event schedule is irregular.
Prediction 4: Signal detection metrics will show a similar patterning of results as related to the previous performance hypotheses. More specifically, participants will have worse perceptual sensitivity and more conservative response bias in the irregular task, for which higher trait self-control will reduce this effect.
Prediction 5: Self-control will decrease task engagement.
Prediction 6: There will be more activation overall for the temporally irregular condition due to the increased difficulty and resource demand of this more challenging condition.
Prediction 7: Event irregularity will produce left-hemispheric prefrontal effects.
Prediction 8: Self-control will decrease activation in dorsolateral prefrontal cortex.
Prediction 9: Self-control will decrease activation in superior parietal cortex.
Prediction 10: Self-control will decrease activation in inferior parietal cortex.

**Table 1:** Table of evidence related to study hypotheses and predictions.

**Prediction**	**Statement**	**Evidence**
P1	The irregular task will result in lower hit rates and higher false alarms than the regular task.	+Evidence for FAs –No evidence for CDs
P2	Individuals higher in self-control will have higher hit rates and lower false alarms in the irregular task.	+Evidence for FAs –No evidence for CDs
P3	Self-control will have a greater moderation on the relationship between time-on-task performance the irregular task as compared to the regular task.	–No evidence for FAs –No evidence for CDs
P4	Signal detection metrics will show a similar patterning as the previous performance hypotheses.	+Evidence for A' in the irregular task +Evidence for A' in the irregular task covarying with self-control +Evidence for c being more liberal in the irregular task –No evidence for B”d
P5	Self-control will cover with changes in task engagement.	+Evidence for self-control decreasing task engagement over time
P6	There will be more activation for the temporally irregular condition.	+Evidence for more activation.
P7	Event irregularity will produce left-hemispheric prefrontal effects.	+Evidence for activation (+HbO2) at lDMPFC.
P8	Self-control will decrease activation in dlPFC.	–No evidence for deactivation at dlPF.
P9	Self-control will decrease activation in rSPL.	+Evidence for deactivation (+HbR) at rSPL.
P10	Self-control will decrease activation in rIPL.	+Evidence for deactivation (+HbR) at rIPL.

## 3 Materials and methods

### 3.1 Participants

Twenty-nine college-age (M = 20.38, SD = 3.21) participants (16 female, 13 male) were recruited from a large, mid-Atlantic university in the United States. Initially, 15 participants experienced regular event schedules while 15 participants experienced irregular event schedules—but one participant from the latter group was removed due to data not recording properly. All participants were compensated for their time and ethically treated according to the guidelines of the American Psychological Association. According to a G-Power 3.1 *post-hoc* power analysis, for an analysis of covariance with our most complex design (numerator df = 3; denominator df = 27), a sample size of 28 is sufficient for our minimum achieved effect size (ηp^2^ = 0.08).

### 3.2 Design

A 2 × 4 mixed-factorial design was used, where participants were randomly assigned to the regular (temporally regular) or irregular (temporally irregular) event schedule condition and time was discretized into four watch periods as a repeated-measures factor.

### 3.3 Tangney, Baumeister, and Boone Self-Control Scale

Trait self-control refers to the personality trait ability to self-override responses and alter personal states or behaviors that are more dominant responses (Baumeister and Alquist, 2009). To study self-control, we used a 36-item measure that examines trait self-control in relation to habit breaking, resisting temptation, and self-discipline (SCS; Tangney et al., 2004). A sample item from the scale reads “I am good at resisting temptation”. The reliability of the scale (ɑ) is 0.89.

### 3.4 Procedure

Participants first completed the self-control questionnaire and the pre-task short stress-state questionnaire (SSSQ; Helton and Näswall, 2015). After a baseline period of 10 min (for rest and neuroimaging purposes), they were randomly assigned to a computerized vigilance task in PsychoPy3 consisting of either irregular or regular event schedules. In both conditions, there was a controlled average inter-stimulus interval (ISI) of 2 s (no fixations present, a blank grey screen). However, the irregular condition involved randomly jittered ISIs (range of 0.6 to 3 s between events), while the regular condition utilized consistent ISIs (2 s between each event). In the irregular condition, ISIs were discretely binned into an inverted-U shaped hazard distribution where occurrences of extremes were least probable—whilst in the regular condition, they consisted of a flat hazard distribution (see Luce, 1986). A 2 × 9 mm horizontal white bar centered against a grey background served as the stimulus, and the stimulus duration delineated whether a signal was critical (125 ms) or neutral (250 ms). Participants were instructed to press the spacebar if a critical signal was present and abstain from responding otherwise. The intent of the duration task was for participants to orient their attention to only the very brief critical stimuli (performing duration judgements). This difficulty was compounded further in the irregular event schedules task, because the experimental manipulation was the regularity of time between the presented signals. Since there was no manipulation of where stimuli were presented on the screen, this effectively means all duration judgements (whether critical or neutral), by definition, could not be estimated as accurately. After a 2-min forced-choice practice block where they received feedback on their performance afterwards (threshold of 70% accuracy required to participate in the experimental trials; maximum of two attempts which all participants completed successfully), they engaged in the 24-min experiment (four blocks of 6 min each), followed by the post-task SSSQ. No feedback was provided during the experiment. Given the fast and continuous presentation of stimuli, participants were simply instructed to respond as accurately as possible. This was done as the temporal pressure was initially likely to make them prioritize speed over accuracy. Average responses were faster than the minimum time between each presented stimulus in the fastest trials (a 600 ms ISI and 125 ms critical signal pair, 725 ms total) in both the regular task (717 ms, SE = 10 ms) and irregular task (674 ms, SE = 10 ms). Additionally, responses were logged in the order they appeared, to account for rare cases in which participants responded after the next stimulus was displayed. Response times were not trimmed, as this may underestimate sampling bias (Panis et al., 2020)—and only response times to correct detections were analyzed. The above procedure and stimuli were taken directly from Shaw et al. (2009, 2012), but with the task instead being reduced from 40 to 24 min in length.

### 3.5 fNIRS procedure

**Figure 1:**
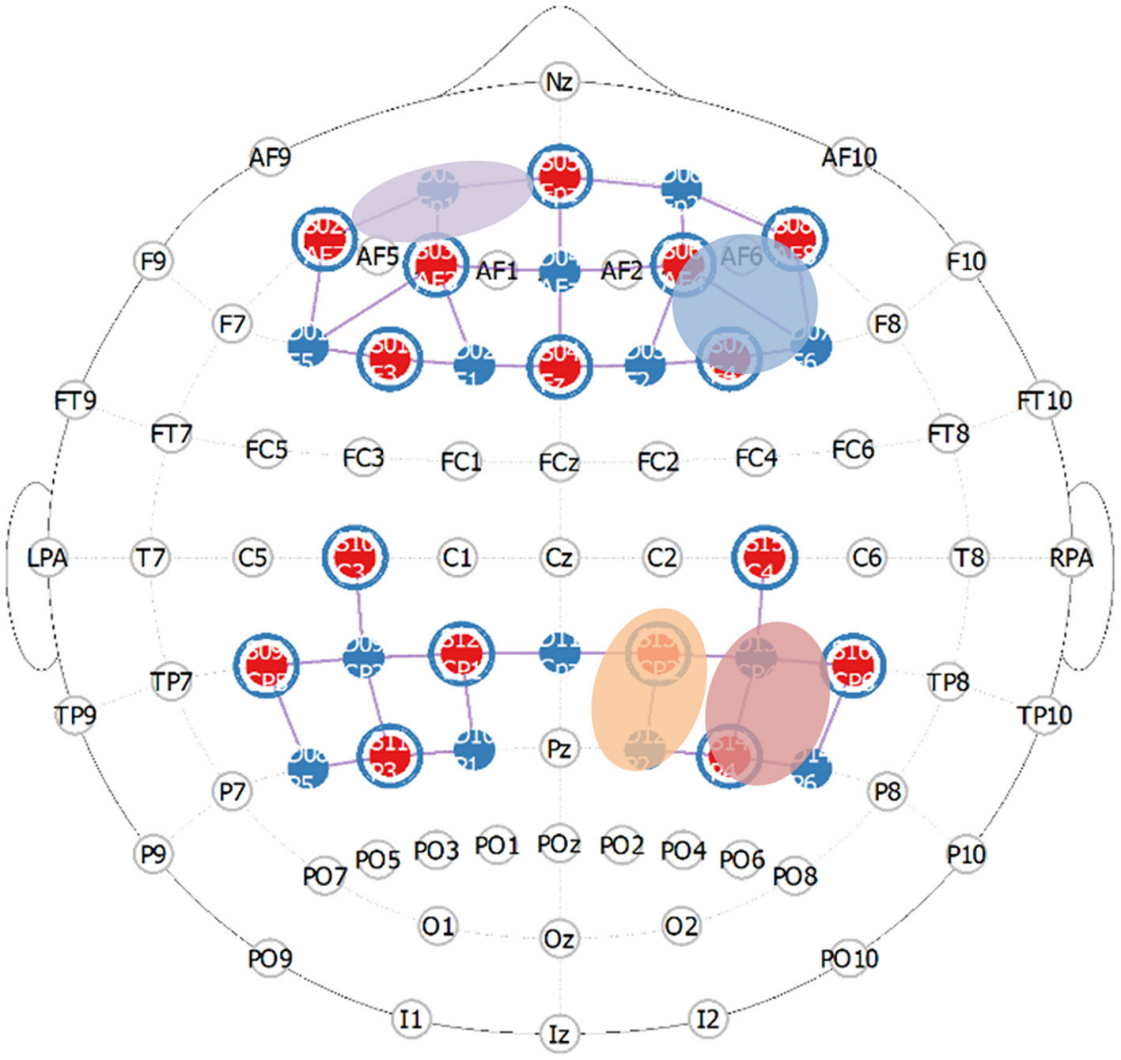
Prefrontal and parietal montage approximating critical regions in the frontoparietal attentional network (purple = lOFC, blue = rdlPFC, orange = rSPL, red is rIPL).

Functional near-infrared spectroscopy (fNIRS) measurements of relative HbO2 and HbR concentrations (850 nm and 760 nm; 10.2 Hz) were recorded during the experiment using two daisy-chained NIRx NIRSport 2 imaging systems (NIRx Medical Technology, Berlin). Participants' head circumferences were recorded using a measuring tape and were then downsized to the nearest appropriate cap size (54, 56, 58, or 60 cm cap). Additionally, the distances between their left- and right-preauricular points as well as between their nasion and inion were recorded. The montage consisted of 46 optodes total, covering both the prefrontal cortex (8 sources, 7 detectors, 8 short-distance detectors) and parietal cortex (same as previous). The montage layout was determined a priori for maximal coverage of our desired Brodmann areas using fNIRS Optodes' Location Decider (fOLD). Then, the montage was manually created in NIRSite (see Figure 1). Optodes were configured according to the international 10–20 system and used a 3 cm long source-detector separation (short-distance channels = 8 mm). The areas of interest included dorsolateral prefrontal cortex (dlPFC; approximating a larger area of the middle frontal gyrus previously mentioned, MFG), dorsomedial prefrontal cortex (dmPFC), orbitofrontal cortex (OFC), superior parietal lobule (SPL; approximating a larger area of the intraparietal sulcus previously mentioned, IPS), inferior parietal lobule (IPL; approximating a larger area of the temporoparietal junction previously mentioned, TPJ).

During fNIRS acquisition, automatic triggers were scripted in PsychoPy3 for the onsets of 32 critical signals (20 s each; 560 total trials) across the 24-min vigilance task. Eight critical signals appeared in each watch period (signal probability = 6%, event rate = 30/min). Due to the theoretical problem of measuring uninterrupted high event-rate vigilance with the hemodynamic response function, we used a slow event-related design consisting of only the critical signals vs. participants' baseline hemodynamic response functions (HRFs). This was done in order to artificially generate “rest” periods for the HRFs but not the participants, as critical signals were highly infrequent compared to the neutral stimuli. Thus, the vigilance task utilized was continuous and break periods were not employed for participants during the experiment.

### 3.6 fNIRS pre-processing

Neural data were pre-processed and analyzed using the NIRS Brain AnalyzIR Toolbox (Santosa et al., 2018). Signals were first down sampled to 5 Hz to reduce autocorrelation, and then corrected for motion outliers and low frequency trends using a temporal wavelet filter (S.D. ≥ 5). Raw voltages were converted to optical densities, and then a spatial PCA filter was used to reduce spatial covariance and further correct motion. Then, optical densities were converted to HbO2 and HbR using the modified Beer-Lambert Law (Cope and Delpy, 1988). Moreover, participants' head circumferences, preauricular distances, and nasion-inion distances were registered to participants' 3-dimensional montage models. They were then warped to a single head model, in order to more effectively localize brain subregions at a group-level while accounting for individual variation in head shape and size. We computed an autoregressive-iteratively reweighted least squares (AR-IRLS) general linear model using the canonical basis HRF, consisting of short-channel (Mayer waves, respiration, cardiac cycles) and accelerometer (yaw, pitch, and roll) nuisance regressors to determine hemoglobin concentrations. FDR-adjusted q-values (instead of *p*-values) are reported here, in order to correct for multiple comparisons across our 30 long-separation channels.

### 3.7 fNIRS design

In the group-level analyses, the same exact regression form of the behavioral ANOVA design was used in order to model the effects of self-control on neural responses in addition to watch period and event regularity. All brain-behavior correlations and neural depictions consist of standardized beta coefficients that survived multiple comparisons adjustments.

## 4 Results

### 4.1 Behavioral results

First, ANOVAs were conducted using the aforementioned design on all relevant behavioral variables. Then, ANCOVAs (with self-control) were conducted to investigate whether trait self-control accounted for any of these effects.

#### 4.1.1 Correct detections

**Figure 2:**
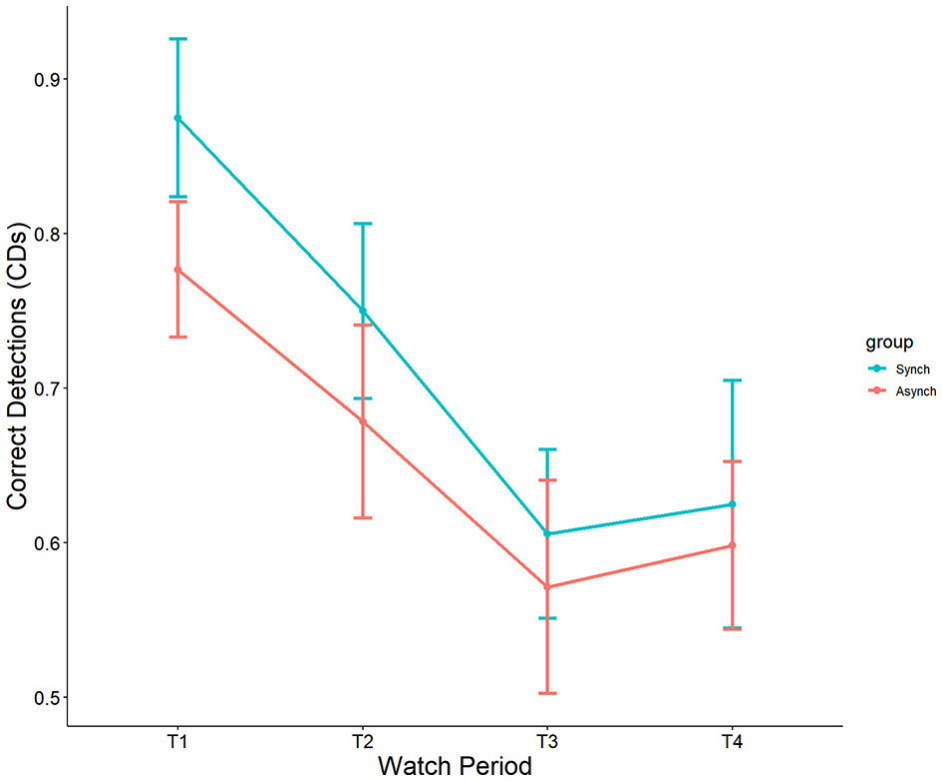
Main effect of watch period on correct detections.

Results indicated a significant main effect of watch period on correct detections, F_(3, 27)_ = 6.38, *p* < 0.001, ηp^2^ = 0.15 (see Figure 2). During the course of the task, correct detections decreased from the first watch period (M = 82%, SE = 4.3%), to the second watch period (M = 71%, SE = 4.3%), up until the third (M = 58.2%, SE = 4.3%) and fourth watch periods (M = 60%, SE = 4.3%). However, no main effect of event regularity (*p* = 0.36, ηp^2^ = 0.008) nor interaction (*p* = 0.92, ηp^2^ = 0.005) was present.

Self-control was not a significant covariate with correct detections in the ANCOVA model, F_(1, 27)_ = 0.89, *p* = 0.35, ηp^2^ = 0.009.

#### 4.1.2 False alarms

**Figure 3:**
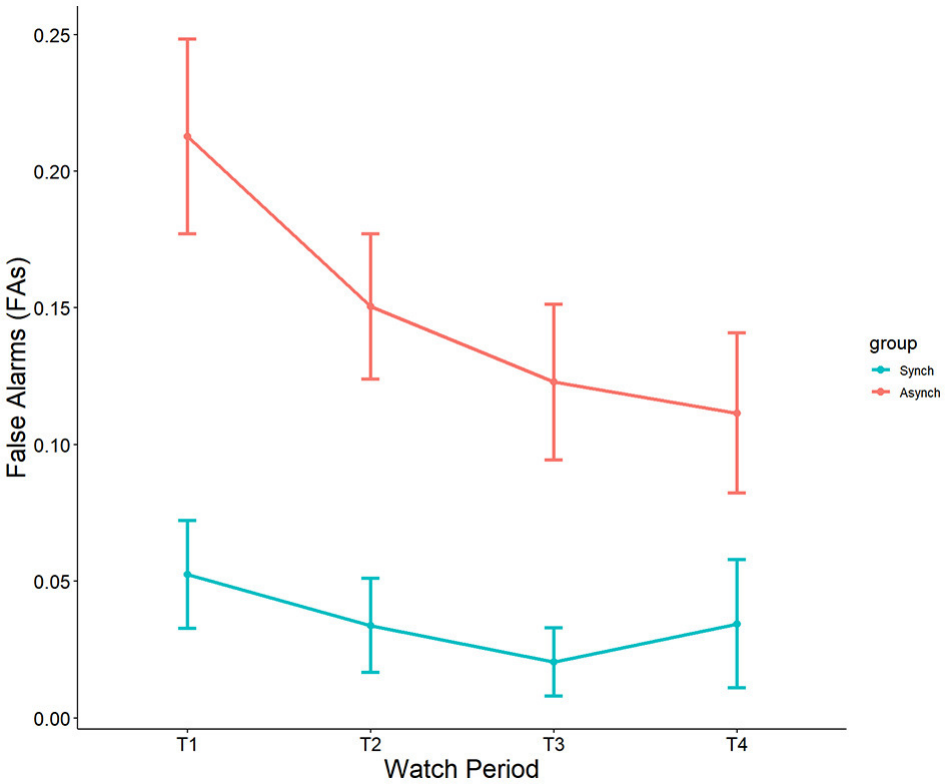
Main effects of watch period and event regularity on false alarm rates.

Results showed a significant main effect of watch period on false alarms, F_(3, 27)_ = 2.90, *p* = 0.04, ηp^2^ = 0.08. During the course of the task, false alarm rates decreased from the first watch period (M = 15%, SE = 1.8%), to the second watch period (M = 10%, SE = 1.8%), up until the third (M = 7.3%, SE = 1.8%) and fourth watch periods (M = 7.6%, SE = 1.8%). Additionally, a main effect was present for event regularity, F_(1, 27)_ = 36.46, *p* < 0.001, ηp^2^ = 0.25. False alarm rates were significantly higher in the irregular (M = 15%, SE = 1.3%) than in the regular (M = 4.3%, SE = 1.2%) condition (see Figure 3). No interaction between watch period and event regularity was present (*p* = 0.47, ηp^2^ = 0.02).

**Figure 4:**
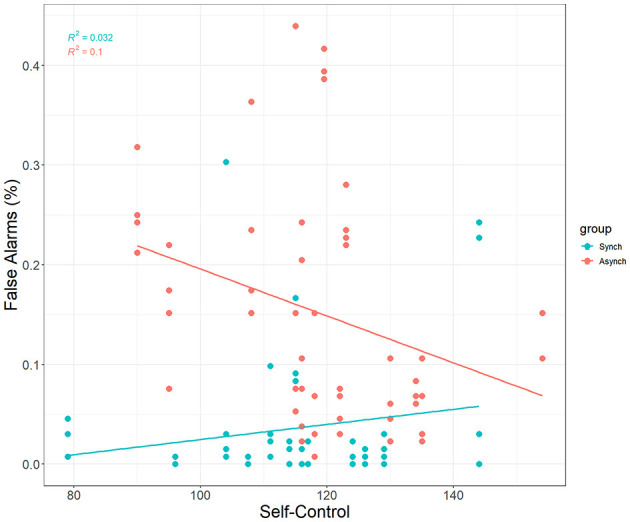
Correlation between self-control and FAs.

Self-control was a significant covariate with false alarms in the ANCOVA model, F_(1, 27)_ = 5.31, *p* = 0.02, ηp^2^ = 0.05. This reveals that self-control does account for differences in false alarms, and that this happens only in the irregular (r = −0.32, *p* = 0.02) task, but not the regular task (r = 0.18, *p* = 0.21) (see Figure 4).

#### 4.1.3 Nonparametric perceptual sensitivity

**Figure 5:**
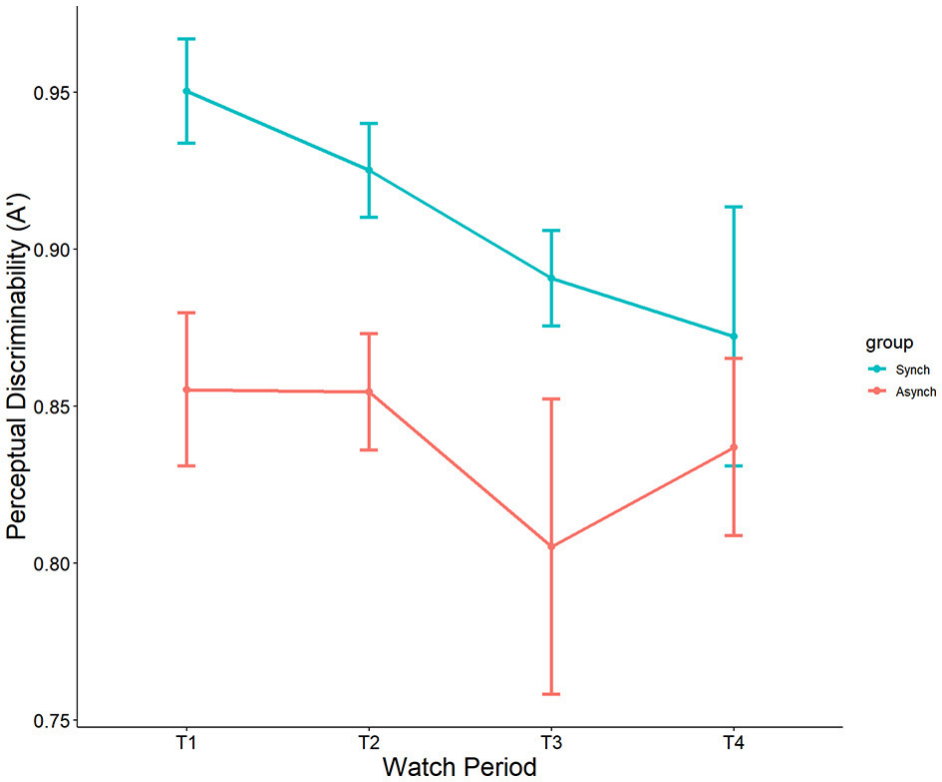
Main effect of event regularity on A'.

We used nonparametric signal detection indices to further examine the data. Results indicated a significant main effect of event regularity on A', F_(1, 27)_ = 11.75, *p* < 0.001, ηp^2^ = 0.10. Perceptual sensitivity was significantly higher in the regular (M = 0.90, SE = 0.013) than in the irregular condition (M = 0.84, SE = 0.014). However, no main effect of watch period (*p* = 0.13, ηp^2^ = 0.05) nor interaction (*p* = 0.69, ηp^2^ = 0.01) was present (see Figure 5).

**Figure 6:**
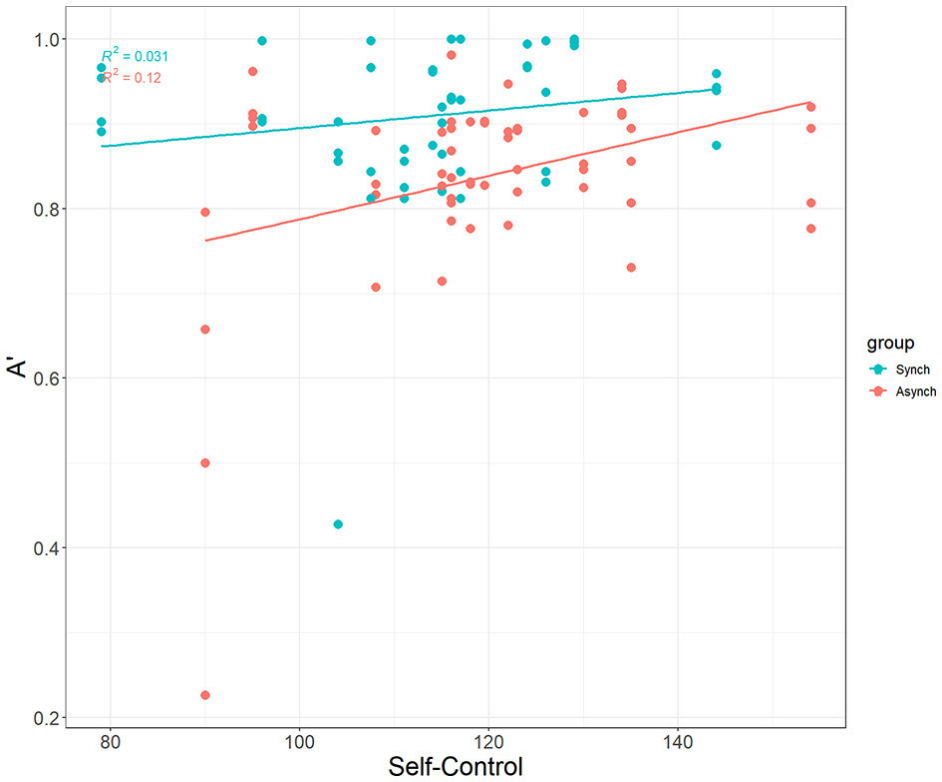
Correlation between self-control and A'.

Interestingly, self-control was a significant covariate with A' in the ANCOVA model, F_(1, 27)_ = 7.49, *p* = 0.007, ηp^2^ = 0.07. This demonstrates that self-control does account for differences in nonparametric perceptual sensitivity, but that this is driven only by the irregular (r = 0.35, *p* = 0.009) task, not the regular task (r = 0.18 , *p* = 0.21) (see Figure 6).

#### 4.1.4 Response bias

**Figure 7:**
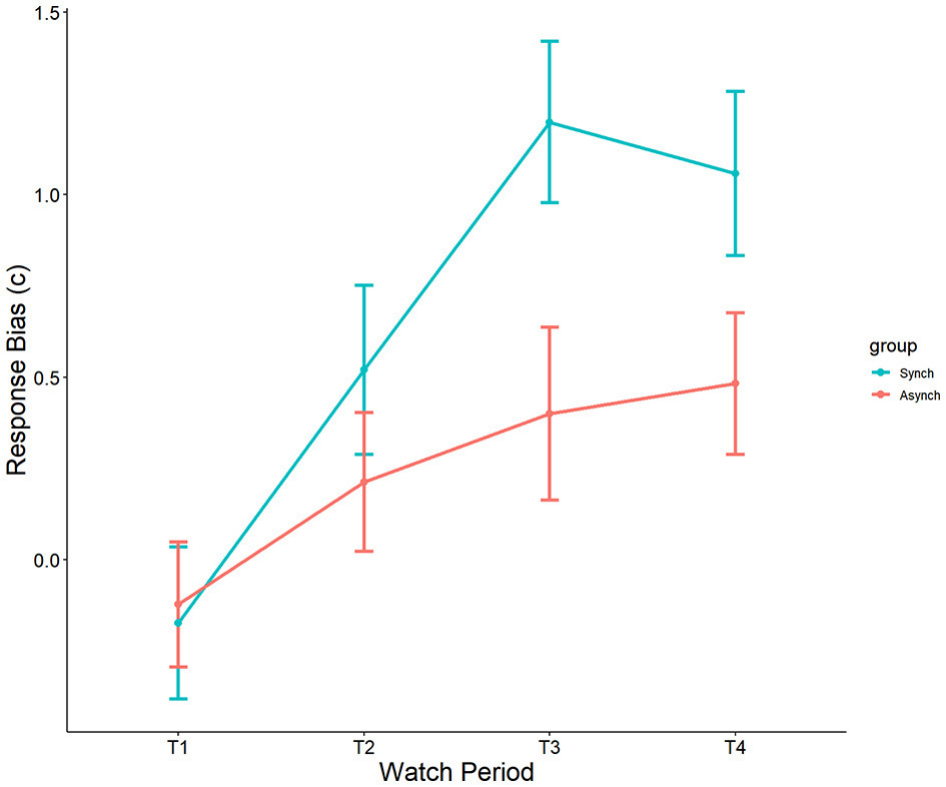
Main effects of watch period and event regularity on c.

Results showed a significant effect of watch period on c, F_(3, 27)_ = 8.56, *p* < 0.001, ηp^2^ = 0.19. During the course of the task, response tendencies became more conservative from the first watch period (M = −0.15, SE = 0.15), to the second watch period (M = 0.34, SE = 0.15), up until the third (M = 0.79, SE = 0.15) and fourth watch periods (M = 0.79, SE = 0.15). Additionally, a main effect was present for event regularity, F_(1, 27)_ = 6.67, *p* = 0.01, ηp^2^ = 0.06. Response bias was significantly more conservative in the regular (M = 0.64, SE = 0.11) than in the irregular (M = 0.24, SE = 0.11) condition (see Figure 7). However, no interaction between watch period and event regularity was present (*p* = 0.23, ηp^2^ = 0.04).

Self-control was not a significant covariate with c in the ANCOVA model, F_(1, 27)_ = 0.02, *p* = 0.88, ηp^2^ = 0.0002.

#### 4.1.5 Nonparametric response bias

**Figure 8:**
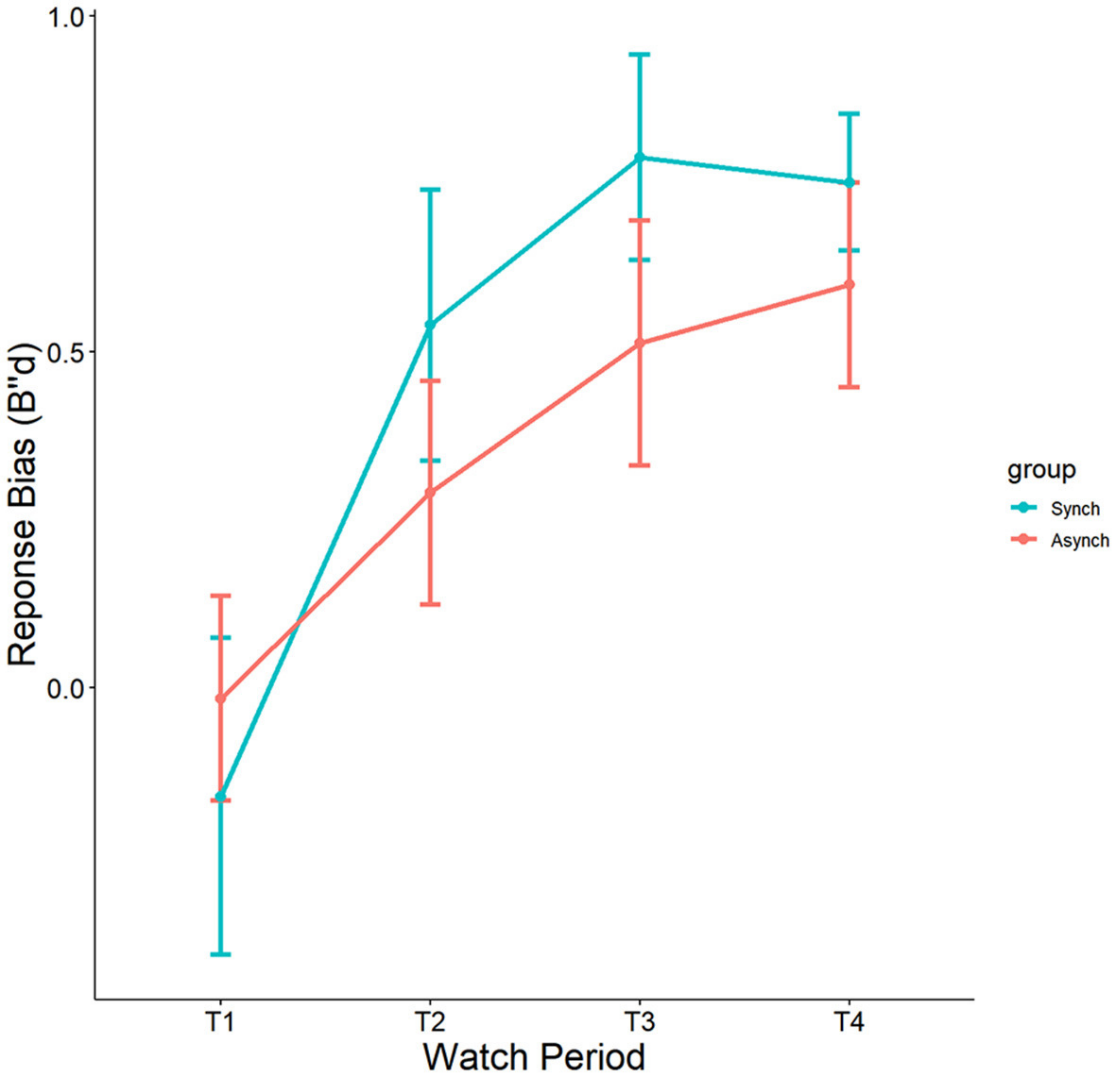
Main effects of watch period and event regularity on B”d.

Results indicated a significant effect of watch period on B”d, F_(3, 27)_ = 8.36, *p* = 0.00005, ηp^2^ = 0.19. During the course of the task, response tendencies became more conservative from the first watch period (M = −0.08, SE = 0.12), to the second watch period (M = 0.38, SE = 0.12), up until the third (M = 0.65, SE = 0.12) and fourth watch periods (M = 0.67, SE = 0.12) (see Figure 8). However, no main effect of event regularity (*p* = 0.35, ηp^2^ = 0.008) nor interaction was present (*p* = 0.69, ηp^2^ = 0.01).

Self-control was not a significant covariate with B”d in the ANCOVA model, F_(1, 27)_ = 0.05, *p* = 0.82, ηp^2^ = 0.0005.

#### 4.1.6 Response time

**Figure 9:**
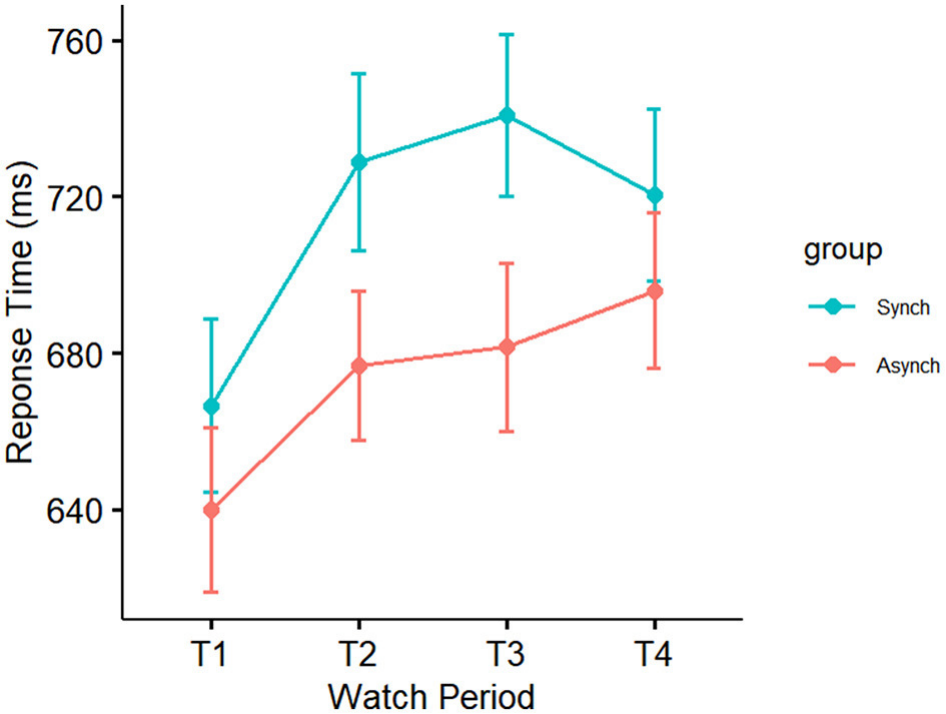
Main effect of event regularity on response time.

Results showed a significant main effect of watch period on response time, F_(3, 27)_ = 3.71, *p* = 0.01, ηp^2^ = 0.10. During the course of the task, response time slowed from the first watch period (M = 654 ms, SE = 14 ms), to the second watch period (703 ms, SE = 14 ms), up until the third (M = 712 ms, SE = 14 ms) and fourth watch periods (M = 711 ms, SE = 14 ms). Results also indicated a significant main effect of event regularity on response time, F_(1, 27)_ = 8.86, *p* = 0.004, ηp^2^ = 0.08. Response time was significantly faster in the irregular (M = 674 ms, SE = 10 ms) compared to the regular condition (M = 717 ms, SE = 10 ms). However, no interaction was present (*p* = 0.79, ηp^2^ = 0.01; see Figure 9).

Self-control was not a significant covariate with response time in the ANCOVA model, F_(1, 27)_ = 0.01, *p* = 0.92, ηp^2^ = 0.0001.

### 4.2 fNIRS results

Mixed effects regressions (random intercept for subjects, random slopes for watch periods) were conducted to investigate the relationship between self-control, task type, and watch period on brain activity. All tests involving watch period used effects coding involving changes over time (period *n* minus period 1). The same design was employed as in the behavioral analyses, but here we account for individual variation in brain activation over time using the following equation:


$$\begin{array}{r} Y=\;‒1+\beta_{1}{\left(Group\right)}_{i}^{*}\beta_{2}{\left(Period\right)}_{i}^{*}\beta_{3}{\left(SelfControl\right)}_{i}\\ +\left(Period\;|\;Subject\right)\;+\;\varepsilon_{0} \end{array}$$

#### 4.2.1 Event regularity

**Figure 10:**
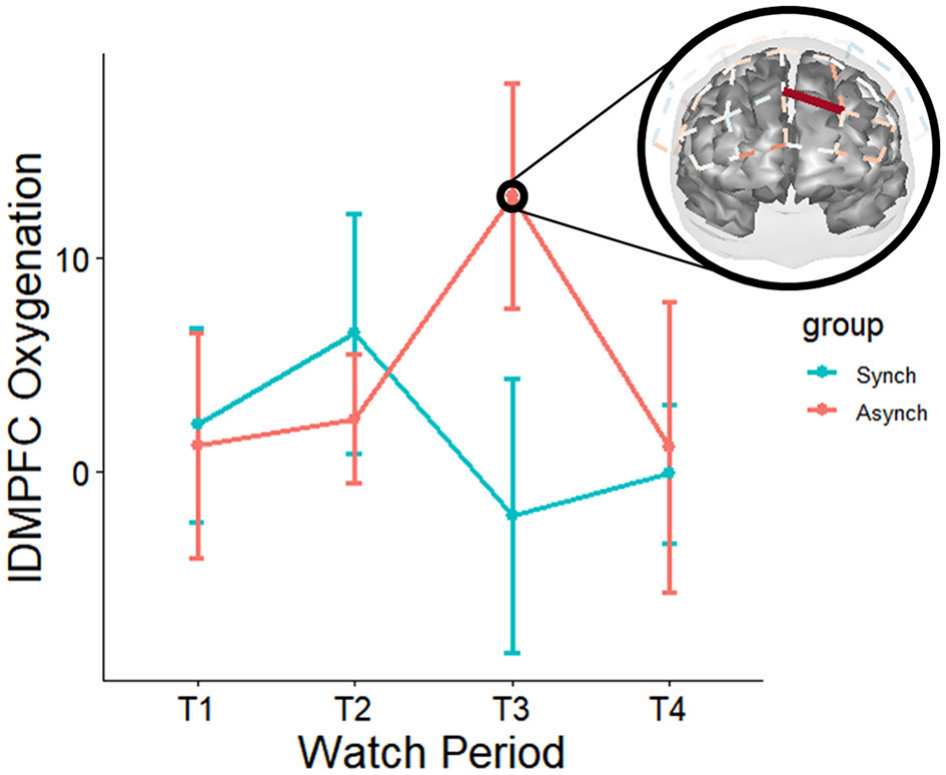
Moderation of neural oxygenation across watch periods for event regularity (red on brain ma*p* = irregular – regular task). Over the course of the experiment, the irregular task increased oxygenation at left dmPFC. Only tests that survived FDR adjustments for multiple comparisons at the q < 0.05 level are depicted. However, it is pertinent to note non-adjusted *p*-values (*p* < 0.05) suggest that the regular task increased oxygenation at right dmPFC early in the task, and over the course of the study decreased oxygenation at right dlPFC at multiple sites (in agreement with previous TCD studies), with the most noticeable trends occurring during watch period 4. These patterns are also supported by non-adjusted *p*-values for deoxygenation—suggesting the highest activation for the irregular task was during period 3, whilst the highest activation for the regular task was during period 4. For these tasks, the trends suggest mirrored effects across the hemispheres between two left dmPFC and two right dmPFC channels, respectively.

Mixed effects regressions (random intercept for participant, random slopes for watch periods) were conducted to investigate whether a two-way interaction between event regularity and watch period was present. Most results for neural utilization (HbR) did not survive multiple comparison-adjusted tests, except for a moderation by event regularity for watch period 1 deoxygenation at the right frontal eye fields (rFEF) for the irregular task, t_(11590, 27)_ = −4.35, q = 0.02. The frontal eye fields are known to be involved in the processing of uncertainty (irregularity), which may be the reason for this finding. In addition, they are known to be activated under periods of active fixation—which may reflect a greater necessity to fixate in the strenuous irregular task (i.e., affording less chances to look away from the flashing stimulus). Most results for neural supply (HbO2) did not survive multiple comparison-adjusted tests. However, there was a moderation by event regularity for changes from watch period 1 to period 3 oxygenation at the left dorsomedial prefrontal cortex (lDMPFC) for the irregular task, t_(11590, 27)_ = 4.49, q = 0.02. This indicates that as time on task progressed, neural supply to the dorsomedial prefrontal cortex increased in the irregular task only. Furthermore, this result agrees with previous studies indicating a left-hemispheric control system for event irregularity (see Figure 10).

#### 4.2.2 Brain-performance correlations

Change scores in performance were correlated with all neural subregions that showed significant (q < 0.05) activation. Performance scores were computed (watch period 3 minus watch period 1) solely based on the behavioral analyses' time on task effects, which indicated that period 1 to period 3 consisted of the greatest changes in performance. Neural deoxygenation is represented in the same manner for the sake of congruency (watch period 3 minus watch period 1), thus constituting joint neurobehavioral changes associated with vigilance.

**Figure 11:**
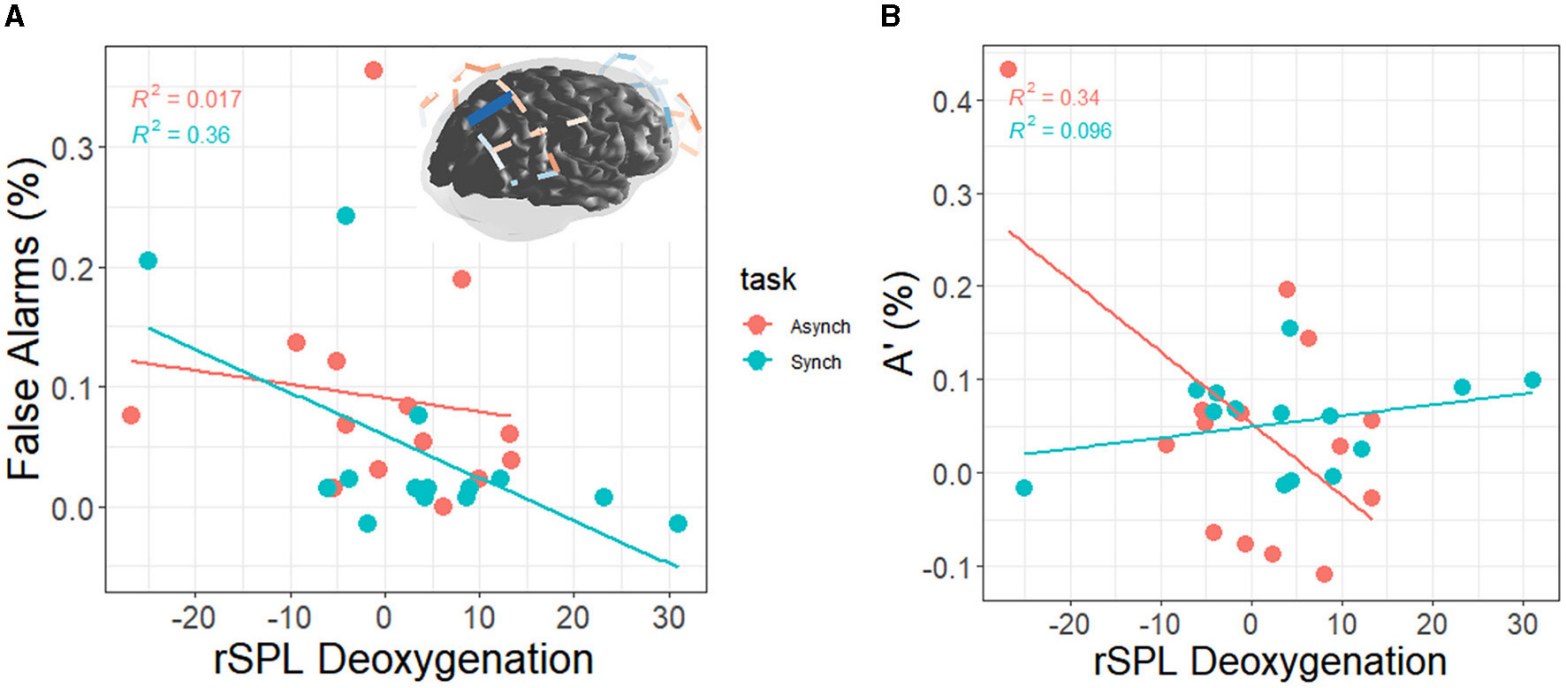
**(A)** Correlation between right superior parietal lobule deoxygenation and FAs (blue on brain ma*p* = +FAs); the values on each axis represent change scores from watch period 1 to watch period 3. From watch period 1 to period 3, activation of rSPL was associated with greater decrement in perceptual sensitivity during the regular task (+FA = decrement, –FA = increment). **(B)** Correlation between right superior parietal lobule deoxygenation and A' (+A' = decrement, –A' = increment). From watch period 1 to period 3, activation of rSPL was associated with greater decrement in perceptual sensitivity during the irregular task.

There was a correlation between right superior parietal lobule deoxygenation and FAs in the regular task (r = −0.60, *p* = 0.02) but not the irregular task (r = −0.13, *p* = 0.66). This demonstrates that individuals with less activation were better able to resist committing false alarms over time than individuals with more activations (see Figures 11A). Finally, there was a correlation between right superior parietal lobule deoxygenation and A' in the irregular task (r = −0.58, *p* = 0.03) but not the regular task (r = 0.31, *p* = 0.28). This demonstrates that individuals with less activation were better able to discriminate between signals and noise over time than individuals with more activation (see Figure 11B).

#### 4.2.3 Trait self-control

**Figure 12:**
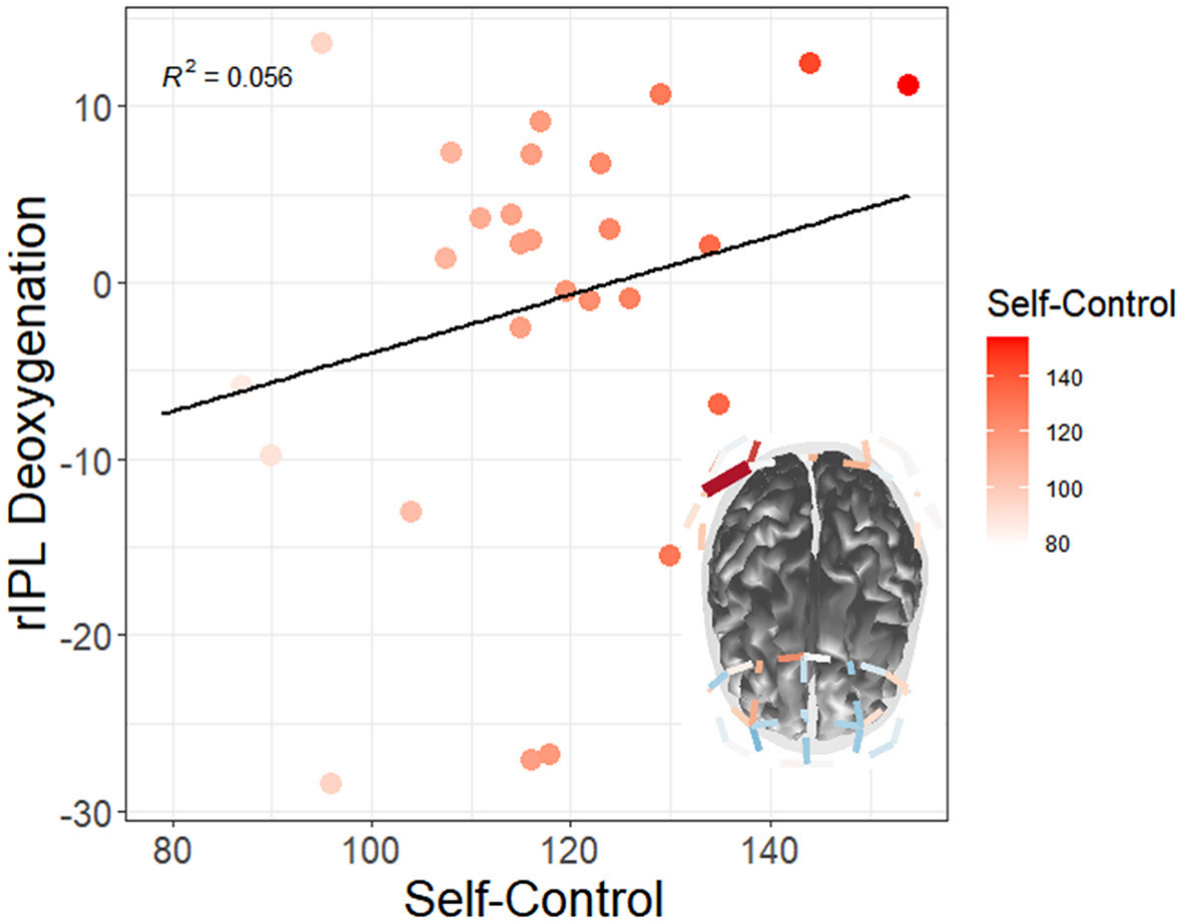
Moderation of watch period by trait self-control on changes in neural deoxygenation (red = high self-control). From watch period 1 to period 3, high self-control appeared to deactivate right inferior parietal lobule (rIPL). Only tests surviving FDR multiple comparisons adjustments at the q < 0.05 level are depicted.

Results for neural supply (HbO2) related to self-control did not survive multiple comparison-adjusted tests. However, results for neural expenditure (HbR) related to self-control did survive adjustment tests. Specifically, there was a moderation by self-control for neural expenditure at left orbitofrontal cortex (lOFC) for changes from watch period 1 to period 2, t_(11195, 27)_ = 3.01, q = 0.02. Furthermore, results showed a moderation by self-control for deoxygenation at right inferior parietal lobule (rIPL) for changes from watch period 1 to period 2, t_(11195, 27)_ = −4.12, q = 0.01. This reveals that directly prior to (but not during) the period of changes in performance, participants low in self-control were expending more neural resources in left orbitofrontal cortex—whereas those high in self-control were instead using more of their right parietal cortex. Lastly, results indicated a moderation by self-control for changes from watch period 1 to period 3 deoxygenation at rIPL, t_(11195, 27)_ = 3.86, q = 0.01. This demonstrates that low self-control individuals were using their right parietal cortex more than their high self-control peers during the period of actual performance changes (see Figure 12).

**Figure 13:**
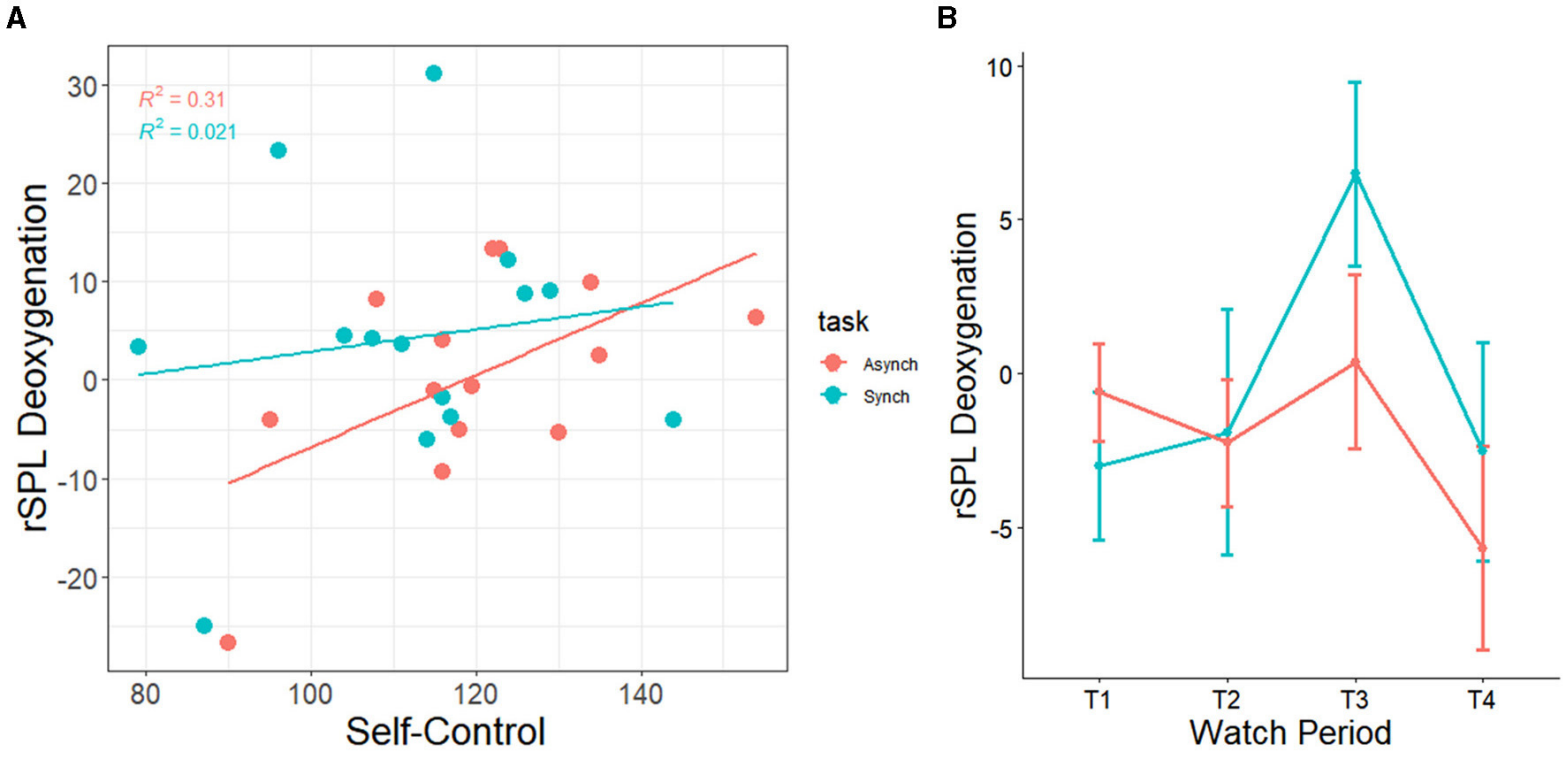
**(A)** Correlation between trait self-control and changes in neural deoxygenation of the right superior parietal lobule. From watch period 1 to period 3, high self-control was associated with deactivation in right superior parietal lobule during the irregular task. **(B)** Depiction of deoxygenation in both tasks over time (-HbR = activation, +HbR = deactivation).

Additionally, there was a correlation between self-control and right superior lobule (rSPL) deoxygenation from watch period 1 to period 3 in the irregular task (r = 0.56, *p* = 0.04) but not the regular task (r = 0.14, *p* = 0.62), for which high self-control individuals had less activation than their low self-control counterparts (see Figure 13A). Similarly, this finding shows that high self-control individuals were using less resources in their right superior parietal lobule as compared to their low self-control counterparts in the temporally irregular task (and see overall deoxygenation across time in Figure 13B).

### 4.3 Short stress-state questionnaire results

**Figure 14:**
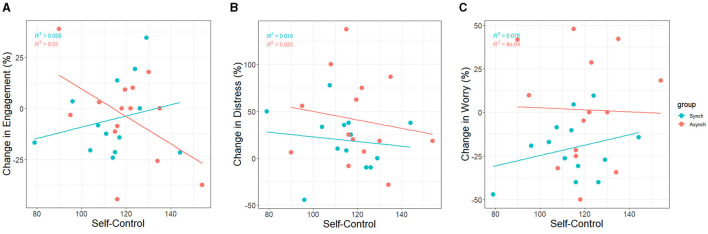
Correlation between trait self-control and standardized changes in **(A)** task engagement, **(B)** distress, and **(C)** worry.

**Figure 15:**
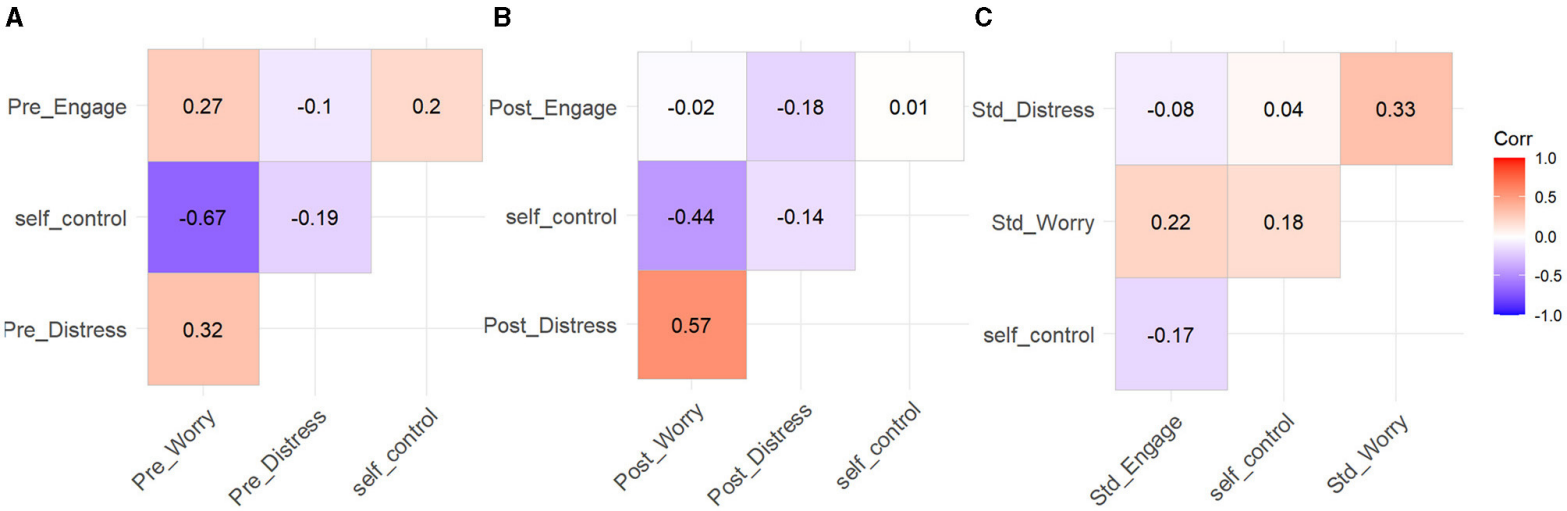
Correlation matrix for self-control, SSSQ pre-task **(A)**, post-task **(B)**, and change scores **(C)**.

Correlations between self-control and pre-task, post-task, as well as standardized change scores (see Figure 14) of engagement, distress, and worry were conducted. Furthermore, correlations were carried out between all variables (see Figure 15). The formula for change scores was:


$$\begin{array}{l} \frac{\left(post‒pre\right)}{pre}\;\times 100 \end{array}$$

#### 4.3.1 Engagement

Baseline scores for engagement were significantly positively associated with post-task engagement (r = 0.53, *p* < 0.001). Furthermore, accounting for task condition showed that this held for the regular task (r = 0.52, *p* < 0.001) and the irregular task (r = 0.54, *p* < 0.001).

At baseline, task engagement was not associated with self-control (r = 0.20, *p* = 0.04). Therefore, individuals high in self-control were no different in their pre-task motivations than their low self-control counterparts. Furthermore, post-task engagement was not associated with self-control (r = 0.01, *p* = 0.89). As such, individuals high in self-control were no different in their post task motivations than their low self-control peers.

Change scores for task engagement were marginally negatively associated with self-control (r = −0.17, *p* = 0.08). Thus, there was only a trend over time overall. However, accounting for task condition revealed that self-control was negatively associated with change scores for the irregular task only (r = −0.50, *p* < 0.001), and marginally associated positively in the regular task (r = 0.24, *p* = 0.09). This indicates that participants high in self-control experienced a greater decline in task engagement in the irregular condition (see Figure 14A).

#### 4.3.2 Distress

Baseline scores for distress were significantly positively associated with post-task distress (r = 0.39, *p* < 0.001). Furthermore, accounting for task condition showed that this held for the regular task (r = 0.52, *p* < 0.001) and the irregular task (r = 0.32, *p* = 0.02).

Baseline scores for distress were not associated with self-control (r = −0.19, *p* = 0.05). This indicates that before the experiment, individuals high in self-control were no different in their distress as compared to their low self-control counterparts. Additionally, post-task distress was not associated with self-control (r = −0.14, *p* = 0.15). This shows that participants high in self-control were no different in their post-task distress than their low self-control counterparts.

Change scores for distress were not associated with self-control (r = −0.09, *p* = 0.37). Therefore, there were no differences between participants high and low in self-control regarding distress over time. Accounting for task condition did not reveal any associations between self-control and change scores in distress in either task (see Figure 14B).

#### 4.3.3 Worry

Baseline scores for worry were significantly positively associated with post-task worry (r = 0.60, *p* < 0.001). Furthermore, accounting for task condition showed that this held for the regular task (r = 0.83, *p* < 0.001) and the irregular task (r = 0.43, *p* = 0.001).

At baseline, worry was negatively associated with self-control (r = −0.67, *p* < 0.001). This shows that before the experiment, individuals high in self-control were less worried than their low self-control counterparts. Furthermore, post-task worry was negatively associated with self-control (r = −0.44, *p* < 0.001). This indicates that after the experiment, individuals high in self-control were less worried than their low self-control peers.

Change scores for worry were not associated with self-control overall. However, accounting for task condition revealed that self-control was positively associated with worry in the regular task only (r = 0.28, *p* = 0.04), not the irregular task (r = −0.03, *p* = 0.83). This indicates that participants low in self-control experienced a greater decline in worrying in the regular condition as compared to their high self-control peers (see Figure 14C).

## 5 Discussion

### 5.1 Performance measures

The present study sought to understand the relationship between trait self-control, temporal regularity, and neural resources expended in relation to vigilance. As is typical in studies of vigilance, a decrement over time was seen across both tasks for correct detections. Why then was a significant decrease in false alarms also observed, as opposed to the traditional increase in false alarms? Crucially, false alarms should not be observed in isolation from response bias in a vigilance activation paradigm where the predominant natural response is withholding a response (96% of the signals were neutral; Lerman et al., 2010). Additionally, in a go/no-go paradigm, participants are instructed to primarily refrain from responding to neutral signals, assigning even greater importance to response inhibition (Raud et al., 2020). In these cases, if response bias is unaccounted for, an increase in false alarms could be spuriously due to indiscriminate responding—whilst a decrease could be due to systematic withholding from responding at the cost of missing critical signals (Lerman et al., 2010). In our study, the latter is indeed the case over the course of the tasks. As response bias became significantly more conservative over time, participants withdrew their attention—leading to less hits and false alarms across the board. However, in the irregular task, response bias was more liberal and participants were actually quicker to respond to stimuli. We suggest this is due to speed-accuracy tradeoffs in vigilance performance (Rubinstein, 2020)—as the irregular task elicited lower perceptual sensitivity and higher false alarm rates from participants. Given that there were so few critical signals, we suggest correct detections were not significantly lower in the irregular task because of a combination of higher false alarm rates, more liberal responding, as well as quickened responding. These findings are consistent with previous research that has explored the signal irregularity effect (Scerbo et al., 1987; Shaw et al., 2012). Self-control predicted false alarms and nonparametric perceptual sensitivity, but did not predict correct detections, response bias, nor reaction time. Additionally, this was only in the irregular task—where responding was more liberal and performance was worse. Therefore, these findings reveal the potential influence of self-control on the vigilance decrement to be primarily related to response inhibition—or, “overriding natural impulses to respond in a more temporally difficult task.”

### 5.2 Resource reallocation hypotheses

In our study we found high self-control to be associated with the deactivation of TPJ (localized more precisely in our study to right inferior parietal lobule; rIPL). This suggests that high self-control participants may not have experienced as much “circuit-breaking,” which allowed them to mitigate neural responses associated with mind-wandering or off-task distractions. Importantly, studies repeatedly demonstrate that mind-wandering frequency can be increased or decreased through modulation of this region (a crucial node in the default mode network; see Kajimura and Nomura, 2015). However, rIPL was not associated with performance in our study. We suggest that this finding aids in clarifying that such mind-wandering processes, while upstream of vigilance functions—do not seem to directly impact vigilance performance in a temporally difficult task. However, this finding does serve as evidence that mindlessness theory still has a role to play in vigilance—as the ability to utilize mental resources is determined by where one “places their resources,” and to what extent they keep them there (resisting circuit-breaking from this region). It may well be the case that a different vigilance task produces performance correlations with the ventral network, and future studies should aim to assess whether there is a mediation between activation and performance via task-unrelated-thoughts (see Scerbo et al., 2005). It is also interesting that the parietal cortex, not the prefrontal cortex—was most sensitive to differences in trait self-control, as there is evidence that self-control is generally associated with PFC activation (e.g., Hare et al., 2009). Here we demonstrate that neural efficiency related to self-control extends to the parietal aspects of both the dorsal and ventral attention networks. Perhaps the ventral attention network primarily involves “peripheral” aspects of vigilance (Vossel et al., 2014). According to those authors, right parietal cortex is causally related to task-switching. Furthermore, as mentioned previously, the ventral attention network can exert a “circuit-break” on the dorsal attention network away from vigilance. Is this happening more in low self-control operators here, given they are using more of their ventral attention networks? In our study, low self-control operators report increases in task engagement—which might be indicative of higher levels of tonic noradrenaline that would keep rIPLactivated. Corbetta et al. (2008) remark that a decrease in tonic noradrenaline causes TPJ to deactivate, which transitions an operator from an exploratory to task-focused state. Conversely, the role of TPJ also serves to disengage from a task (noted by increases in activation due to increases in tonic noradrenaline).

### 5.3 Resource utilization hypotheses

The second critical component of our narrative is the dorsal attention network. On its own, the dorsal attention network represents factors “central” to vigilance. In our study, we found increased efficiency of the inferior parietal lobule (rIPL) and superior parietal lobule (rSPL) in relation to self-control. However, there is an important caveat here. Unlike the former region, rSPL predicted the level of vigilance performance as measured by false alarms and nonparametric perceptual sensitivity(with increases in activation leading to vigilance decrement). Therefore, while self-control may not be as associated with prefrontal functions, associated changes in performance seem to arise from the same parietal aspect of the dorsal attention network—downstream from the influence of rIPL. It is important to note that high self-control and dorsal network optimization were each enough to resist changes in performance over the course of the vigilIn order to understand the significance of each of these nodes within the “circuit-breaking” narrative, Painter et al. (2015) provide a double dissociation of their functions. In their experiment, they used transcranial magnetic stimulation (TMS) to “shut off” either the right IPS or right TPJ—the same regions we studied. They found that rTPJ was only involved in orienting to and from distracting cues (distractor suppression), whereas rIPS was only involved in attentional capture (maintaining vigilance). Therefore, while the efficiency of rTPJ is specific to mitigating mind-wandering—the efficiency of rIPS is specific to maintaining attention. In our study, the noted correlation of performance only with rIPS extends the resource theory narrative such that “vigilance is hard work for some operators” (Warm et al., 2008) as opposed to being a cognitively non-demanding phenomenon. Furthermore, we suggest that the mind-wandering aspect is not predictive of time-on-task changes in performance, but rather the natural transition “in and out” of vigilance. Perhaps mind-wandering could even be mobilized in low self-control operators as a strategy for interleaving attention more effectively (Sana et al., 2018)—as their overutilization of rIPL did not produce consequences.

### 5.4 fTCD vs. fNIRS research

The reason self-control was not significant in fTCD studies may be evident in the way fNIRS constructs depart from those studies. We demonstrate here that task event regularity impacted the flow of blood to left prefrontal areas (supporting the present literature). fTCD relies on the velocity of the blood flowing to cortical regions, which is directly related to the amount of flow. In our study, we measured the latter concept (HbO2), thereby validating those fTCD findings. Why then, did we find self-control effects when those same studies did not? The answer might be that incoming blood flow changes are negligible in the case of trait self-control—because the neural control strategies are operating at the level of energy expenditure(HbR), not incoming resource supply. fTCD does not discriminate between these two concepts, but we are able to disentangle the two notions using fNIRS. Additionally, we are able to delineate differences in myriad neural regions—thus revealing more of a parietal as opposed to prefrontal focus for the vigilance decrement and self-control. Lastly, we are able to provide specific evidence for resource theory and mindlessness theory based on their supposed underlying brain networks.

### 5.5 Stress-state measures

High self-control was also associated with decreases in task engagement, which may reflect perceptions of “lower energetic costs” as compared to the low self-control sample. Such a finding may arise from the fact that less somatosensory resources were used in the former group, as the parietal cortex dictates the integration of visuomotor planning information and activation is known to be positively associated with feelings of fatigue. Through this view, such action planning circuits may become “overloaded” in a difficult vigilance task depending on one's aptitude (self-control), resulting in more false alarms and decreased signal discrimination ability. This is further evidenced by the result that only the irregular task was driving a strong association between self-control and drops in task engagement. Indeed, Helton and Russell (2010) remark that task engagement may index “the amount of resources allocated to the task,” but that claim was in the context of correct detections and response times. We note that in our study, those low in self-control are not suffering from a lack of correct detections nor slower response times—which might in part be due to either indiscriminately pulling the trigger on critical signals, or a disaffected ability of response excitation. Therefore, within our study's context we suggest trait self-control instantiates the “capability to not spend resources recklessly” at both a neural and behavioral level. On the other hand, self-control was not related to changes in distress, nor participants' pre- or post-task scores—suggesting no potential regulatory influences on their affect. However, self-control was highly associated with pre-task worry, post-task worry, as well as changes in worry. High self-control operators were less worried before and after the study than their low self-control counterparts, suggesting there may be some benefit for self-efficacy as it relates to the task (i.e., cognitive appraisals).

### 5.6 Limitations and future work

This study has several limitations. One is that a decrease in task engagement is typically not associated with better task performance. However, many studies studying task engagement do not divide the sample based on self-control, which is a variable inherently related to one's ability to deal with life stressors (i.e., resilience and grit). Additionally, our high self-control participants were much lower in their worry (at baseline and after the task) compared to the low self-control sample, suggesting a low basal level of worrying (as well as resistance to doing so throughout the task) and a decrease in feelings of engagement could typify the “highly self-regulated operator.” In addition, their decreased resource utilization might be related to either of those feelings, though future studies are needed to investigate this specific hypothesis. Our collective findings speak to the necessity to better understand the frontoparietal networks of vigilance in real-world contexts—where previously we have predominantly used frontal regions as indicators of mental resources in vigilance.

### 5.7 Conclusion

In identifying the specific prefrontal subregions impacted by different types of vigilance tasks, we sought to expand on the efforts of prior TCD research. Additionally, we localized decrement-specific effects (and those related to self-control) to areas of the posterior parietal cortex, within the frontoparietal attention networks. This is of critical importance to understanding the mechanism of action that “mental resources” operate by—which is our implicit, underlying objective roots in psychophysiological research on attention. Furthermore, we expand on the individual differences literature to track a broad personality trait (self-control) to those networks, and crucially, to its ability to resist the myriad impacts of vigilance on a human operator. Finally, we elucidated a trait-brain-performance-state relationship between self-control, parietal subregions, the vigilance decrement, and stress states respectively.

## Data Availability

The original contributions presented in the study are included in the article/Supplementary material, further inquiries can be directed to the corresponding author.
